# The Unfolded Protein Response: At the Intersection between Endoplasmic Reticulum Function and Mitochondrial Bioenergetics

**DOI:** 10.3389/fonc.2017.00055

**Published:** 2017-04-03

**Authors:** Amado Carreras-Sureda, Philippe Pihán, Claudio Hetz

**Affiliations:** ^1^Center for Geroscience, Brain Health and Metabolism, Faculty of Medicine, University of Chile, Santiago, Chile; ^2^Biomedical Neuroscience Institute, Faculty of Medicine, University of Chile, Santiago, Chile; ^3^Program of Cellular and Molecular Biology, Institute of Biomedical Sciences, University of Chile, Santiago, Chile; ^4^Buck Institute for Research on Aging, Novato, CA, USA; ^5^Department of Immunology and Infectious Diseases, Harvard School of Public Health, Boston, MA, USA

**Keywords:** mitochondria-associated membranes, unfolded protein response, endoplasmic reticulum stress, mitochondria, cancer

## Abstract

Endoplasmic reticulum (ER) to mitochondria communication has emerged in recent years as a signaling hub regulating cellular physiology with a relevant contribution to diseases including cancer and neurodegeneration. This functional integration is exerted through discrete interorganelle structures known as mitochondria-associated membranes (MAMs). At these domains, ER/mitochondria physically associate to dynamically adjust metabolic demands and the response to stress stimuli. Here, we provide a focused overview of how the ER shapes the function of the mitochondria, giving a special emphasis to the significance of local signaling of the unfolded protein response at MAMs. The implications to cell fate control and the progression of cancer are also discussed.

Cellular organelles are no longer conceived as isolated entities with defined and unique functions, but as dynamic signaling nodes, where a single organelle may engage and influence the functioning of several cellular compartments and processes. Interorganelle interactions are facilitated by specialized structures that tie them together structurally and functionally. Mitochondria-associated membranes (MAMs) are subdomains that bring the endoplasmic reticulum (ER) and mitochondria into close proximity, enabling a complex cross talk (1). This physical association shapes mitochondrial morphology and dynamics (2), in addition to participate in the response to various stress stimuli, modulating metabolism, redox control, and apoptosis.

The ER is the primary site where transmembrane and secretory proteins are folded; in addition to operate as the main intracellular calcium reservoir and a site of lipid biosynthesis. Abnormal accumulation of misfolded proteins within the ER lumen may result in the loss of proteostasis, a condition referred to as ER stress (3, 4). ER stress is triggered by physiological demands including high secretory activity, in addition to pathological conditions that may perturb protein folding and maturation, calcium homeostasis, redox balance, among other events. Under ER stress the unfolded protein response (UPR) is engaged, operating as a dynamic signaling network that enforces adaptive programs to restore proteostasis by reducing the load of unfolded proteins through the upregulation of genes involved in almost every aspect of the secretory pathway (5). However, if ER homeostasis cannot be restored, the UPR switches its signaling toward a proapoptotic mode to eliminate irreversibly damaged cells. Thus, the UPR is a central adjustor to control cell fate under ER stress, contributing to diverse pathological conditions including cancer, neurodegeneration, and diabetes, among others (6).

## The UPR

**Figure 1:**
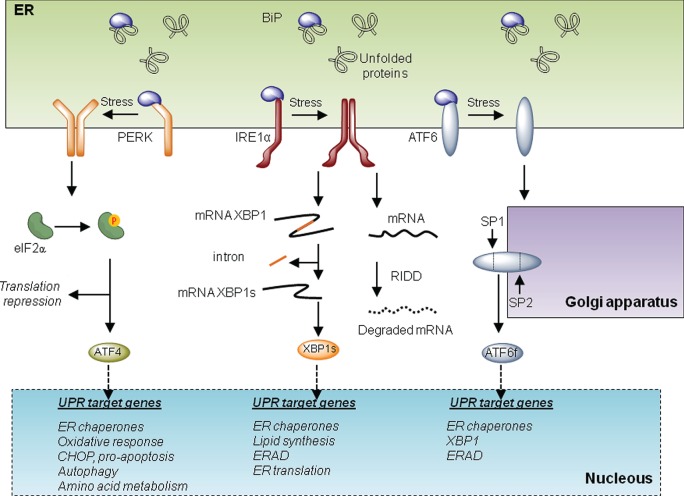
**The unfolded protein response (UPR)**. Three main signaling branches form the UPR. Under resting conditions, BiP protein binds to and inhibits the triggering of the UPR. Under endoplasmic reticulum (ER) stress, BiP dissociates from the UPR transducers to chaperone misfolded proteins in the lumen of the ER. This disassemble promotes the activation of the three branches of the UPR. On the one hand, PKR-like ER kinase (PERK) oligomerizes and phosphorylates eIF2alpha. This phosphorylation provokes a repression of global translation and facilitates the expression of specific transcripts. Among them, activating transcription factor 4 (ATF4) drives the transcription program of the PERK branch that activates genes involved in folding, oxidative responses, autophagy, amino acid metabolism, and apoptosis *via* CHOP. Upon activation, IRE1 oligomerizes and processes the mRNA encoding for X-box binding protein 1 (XBP1), a transcription factor that activates cellular programs involved in ERAD, ER translation, ER chaperones, and lipid synthesis. Finally under stress, activating factor 6 (ATF6) translocates to the Golgi where it is processed by SP1 and SP2 generating a transcription factor that activates UPR target genes involved in ERAD and folding.

The UPR is initiated by at least three distinct ER-localized stress sensors: inositol-requiring enzyme 1 (IRE1α), PKR-like ER kinase (PERK), and activating factor 6 (ATF6). IRE1α is a functional kinase, and RNase represents the most conserved branch of the UPR. Upon activation, IRE1α catalyzes the unconventional splicing of X-box binding protein 1 (XBP1) removing a 26-nucleotide intron. This processing event changes the open reading frame of the mRNA, resulting in the translation of a potent transcriptional activator termed XBP1s (for the spliced form). XBP1s upregulates several genes involved in the UPR’s adaptive phase, having a crucial role in the maintenance of the function of highly secretory cells (7). IRE1α also degrades several mRNA and microRNAs, an activity known as regulated IRE1-dependent decay or RIDD (8), impacting diverse processes including inflammation, stress mitigation, and apoptosis. Activation of PERK leads to the phosphorylation of the eukaryotic translation initiation factor eIF2α, resulting in global protein synthesis arrest reducing ER load (5). Under these conditions, activating transcription factor 4 (ATF4) is differentially translated, upregulating genes involved in protein folding, amino acid metabolism, autophagy, and redox homeostasis. Upon sustained ER stress, ATF4 also contributes to apoptosis through the induction of C/EBP homologous protein CHOP and by enhancing oxidative stress and protein synthesis (4). Finally, ATF6 is retained at the ER under basal conditions but shuttles to the Golgi apparatus under ER stress, where it is cleaved by SP1 and SP2 proteases. This event leads to the release of ATF6 N-terminal fragment, a potent transcription factor that—together with XBP1—regulates the expression of several genes involved in reestablishing ER homeostasis (Figure 1). Overall, depending on the duration and intensity of the stress, the UPR engages different cellular outputs to sustain cell survival or trigger apoptosis. For this balance, the communication between the ER and mitochondria is emerging as an important contributor to cell death under stress, in addition to providing metabolic advantages during early adaptive responses. In this article, we focus in addressing the specific activities of the UPR at MAMs and the implications to mitochondrial physiology. The possible impact to pathological conditions such as cancer is also discussed.

## The UPR and MAMs

MAMs are specialized ER membranes in close proximity to the mitochondria outer membrane, that facilitate the communication between these two organelles (9). In the last decade, this subdomain has emerged as a signaling platform, playing critical functions in lipid biosynthesis, ER to mitochondria calcium transfer, bioenergetics, autophagy, and cell death (10, 11).

The composition and abundance of mammalian MAMs is highly dynamic, shaped by metabolic demands and cellular insults. For example, the tethering between these two organelles is enhanced under ER stress, together with a redistribution toward the perinuclear area (12). Interestingly, the dynamic assembly of MAMs occurs during the early phase of the UPR, which is classically considered to be prosurvival, and correlates with increased mitochondrial calcium uptake and enhanced respiration (12). In mouse models of diabetes—where ER stress is chronically active and MAMs are augmented—experimental manipulation of MAMs’ formation restores glucose homeostasis (13). Along these lines, different pathologies of the central nervous system with a strong ER stress component (14) also develop alterations in MAMs either at the morphological or biochemical level (15). Thus, under acute or chronic ER stress, there is an abnormal cross talk between the ER and mitochondria that may drive pathological events impacting metabolic function, redox balance, and cell death control.

**Figure 2:**
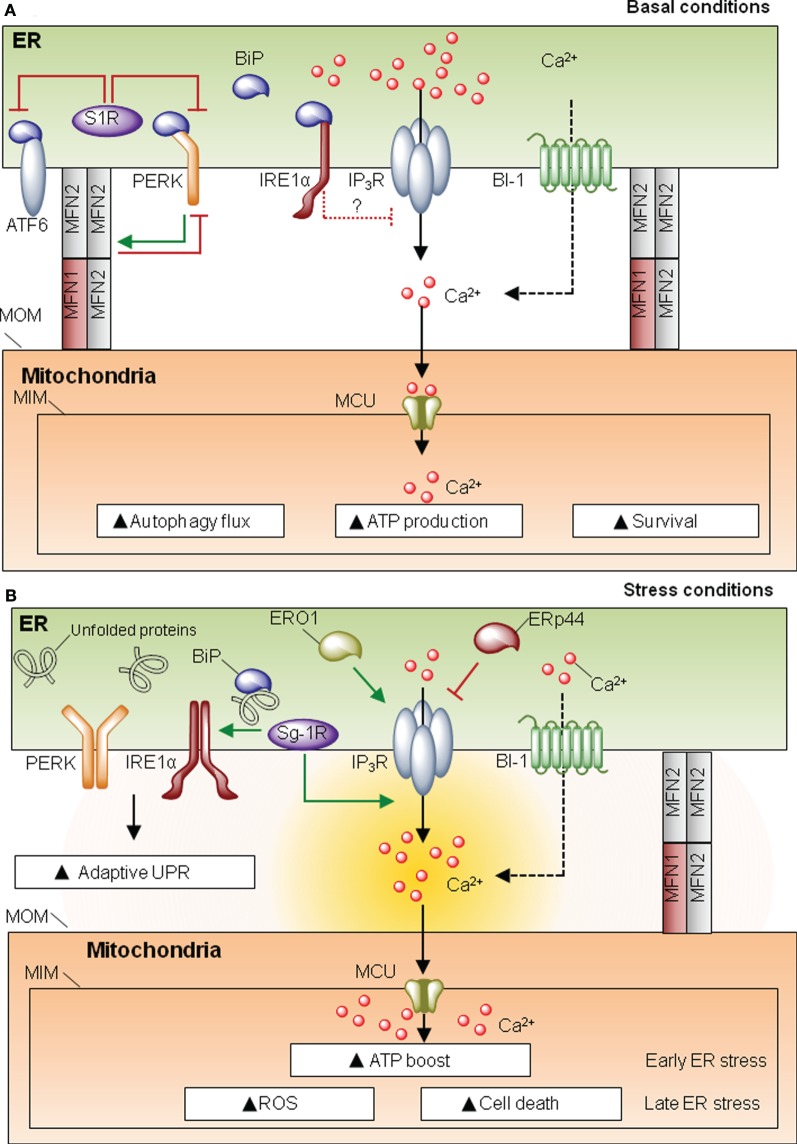
**Mitochondria-associated membranes (MAMs) and the unfolded protein response (UPR)**. **(A)** Under resting conditions, sigma one receptor (S1R) prevents activating factor 6 (ATF6)/PKR-like endoplasmic reticulum (ER) kinase (PERK) activity. IRE1 negatively regulates IP_3_ receptor (IP_3_R) activity. This homeostatic equilibrium is needed to maintain cellular respiration, survival ATP generation, and protein folding. **(B)** Under ER homeostasis disturbances, UPR stress sensors are activated. S1R promotes IP_3_R activity and may stabilize IRE1 RNase function. ERO1 is also promoting IP_3_R activity, whereas ERp44 depending on the PH and redox state will inhibit the activity of IP_3_R type 1, whether this co-occurs in MAMs has still to be directly defined. Under ER stress, there is a convergence for the pro-adaptative phase of the UPR and an ATP boost, due to enhanced calcium entry to the mitochondria *via* mitochondrial calcium uniporter (MCU). However, if the stress levels are not resolved, the UPR shifts its signalling toward a proapoptotic response.

Proteins present in MAMs can be classified as spacers (i.e., increase distance between ER and mitochondria), tethers (i.e., increase contact site formation), or functional components that are not directly related to morphological features. One of the most characterized proteins involved in MAM formation is mitofusin-2 (MFN2), a player first discovered for its role in mitochondria fusion and fission (16). MFN2 is involved in ER–mitochondria interactions despite the fact that its actual function as a tether or spacer is still debated (17–20). MFN2 also modulates ER homeostasis, since cells deficient for this protein develop spontaneous ER stress as demonstrated in cell culture and *in vivo* studies (21–23). The UPR can be engaged when protein folding is compromised due to alterations in ER chaperones. In MAMs there is a relevant set of chaperones and oxidoreductases with functions associated to ER stress (Figure 2). One of the most studied MAM-located chaperones is the sigma one receptor (S1R), a protein implicated in neuroprotection, carcinogenesis, and neuroplasticity (24). Interestingly, S1R acts directly on the three UPR transducers. For example, one study proposed that S1R inhibits PERK and ATF6 signaling (Figure 2A), but it can stabilize the RNAse activity of IRE1 at MAMs (25). Moreover S1R expression is induced under ER stress (26), enhancing the activity of IP_3_ receptor (IP_3_R) (24, 25) (Figure 2B). These observations suggest a clear role for S1R at MAMs, impacting ER physiology, by controlling ER calcium homeostasis *via* IP_3_R, or through the modulation of the UPR signaling. In a similar way, the ER chaperone calnexin (CNX) regulates the activity of sarco/endoplasmic reticulum calcium-ATPase 2b (SERCA2b) (27), and it is enriched in MAMs by two possible mechanisms. When palmitolylated, CNX localizes to MAMs, a modification that is lost under early ER stress responses (28). Additionally, phosphofurin acidic cluster sorting protein 2 (PACS-2) is an integral MAM component that contributes to oxidative folding at the ER (29) and binds to and retains phosphorylated CNX at this membrane subdomain (30). Whether SERCA2b is also present in MAMs and directly interacts with CNX in this structure has not been directly addressed. Importantly, in addition to classical chaperones, different ER oxidoreductases and foldases are present at MAMs, including ERO1α and ERp44 (31) (Figure 2). Similar to S1R, ERO1α also enhances IP_3_R activity contributing to ER stress-mediated cell death and mitochondrial calcium overload (32, 33). In this direction, the ER foldase ERp44 is considered to be present at MAMs since it binds to IP_3_R (34, 35); however, no direct evidence for a function of ERp44 in MAMs has been yet reported. Finally, Bax-inhibitor-1 (BI-1), an evolutionary conserved ER-localized protein with wide roles in apoptosis regulation (36), is also located at MAMs, regulating mitochondrial calcium uptake and apoptosis (37). BI-1 has been shown to repress the UPR and regulate autophagy under stress conditions, through the inhibition of the IRE1α signaling pathway and the modulation of calcium signaling (38–40) (Figure 2). BI-1 has been linked to influence calcium signaling at MAMs; however, whether the regulatory role of this protein over autophagy and the UPR takes place at MAMs is unknown. Overall, the fact that different foldases, chaperones, and oxidoreductases are present at MAMs emphasizes the relevance of this signaling node to engage adaptive programs to sustain cell function under proteostatic stress.

UPR stress sensors may have important biological functions at MAMs, influencing mitochondrial physiology. Core components of the UPR such as PERK and IRE1α have been spotted at MAMs. Under ER stress conditions, IRE1α becomes enriched in MAMs, where it is stabilized, making cells more resistant to ER stress-induced cell death. This stabilization may be mediated by the S1R chaperone (25). Moreover, indirect evidence in neuronal cell lines suggests that IRE1α may play a role in the regulation of ER to mitochondria calcium transfer under basal and ER stress conditions, through the negative modulation of IP_3_R and its coupling with the mitochondrial calcium uniporter (41) (Figure 2).

PKR-like ER kinase is also enriched in MAMs, and PERK-deficient cells have decreased number of ER–mitochondria contact sites and perturbed ER calcium signaling (42). PERK signalling is required for ROS production, sensitizing cells to apoptosis (42). However, it is not clear from this study whether the enhancement of ROS levels by PERK deficiency is the result of its known role in the antioxidant response through the transcriptional activity of ATF4 (43). In contrast, another study suggested that PERK has a protective role at MAMs (21). Surprisingly, MFN2 was shown to operate as an upstream regulator of PERK, preventing its activation through a physical interaction, controlling cell death and mitochondrial morphology (21) (Figure 2). Although it was suggested that PERK might influence MAMs’ abundance and function through the MFN2 axis, the role that MFN2 plays at this compartment is still debated: it may contribute to tethering (18, 20) or operate as a spacer (17, 19). Thus, increasing evidence suggest that the UPR may operate at the structural and functional intersection between the ER and mitochondria to regulate both adaptive and chronic ER stress responses.

## MAMs, UPR, and Cancer

Functional alterations to MAMs may impact cell function and viability resulting on disease conditions, such as cancer. Due to the broad impact of MAMs as a site of control of the UPR and mitochondrial function, it is predicted that MAMs may impact cancer progression by influencing tumor proteostasis and bioenergetics. The most studied output of MAMs in relation to cancer is the regulation of autophagy [reviewed in Ref. (44)] and the transfer of calcium from the ER to mitochondria (45). Mitochondrial calcium uptake not only alters the threshold to induce apoptosis (45) but also fine-tunes metabolism through the regulation of the tricarboxylic acid cycle and the electron transfer chain. It also enhances catabolic processes such as autophagy mediated by the AMP kinase (46, 47), a sensor essential for the growth of tumors. It is noteworthy that different oncogenes and tumor suppressors may influence MAMs (48), with relevant implications to cell survival and malignant transformation. Alterations to ER calcium content have been reported during tumor progression (49), which may modify ER to mitochondria calcium transfer. Moreover, the deregulation of the BCL-2 family in cancer cells may also alter ER calcium content through its known role in fine-tuning the activity of IP_3_R (50).

Mitochondrial energy production is critical to maintain the large energetic demands of certain types of cancer cells that rely on oxidative phosphorylation. This metabolic process is maintained by a tight regulation of calcium transfer from the ER to mitochondria (46). However, excessive mitochondrial calcium overload may result in cell death through the opening of the permeability transition pore (PTP) or by sensitizing mitochondria to canonical intrinsic apoptotic signals (45, 48). In addition, the UPR has been extensively related to cancer due to its relevance to promote cell adaptation to the hypoxia conditions observed in solid tumors (51, 52). Cell adaptation to hypoxia, together with limited oxygen availability, may facilitate the Warburg effect, whereby cancer cells rely on anaerobic glycolysis instead of oxidative phosphorylation. It is interesting to mention that one putative target of the RNAse domain of IRE1 is the mitochondrial pyruvate carrier, a limiting step for the import of pyruvate to mitochondria that regulates the Warburg effect (53). In fact, genetic and pharmacological evidence have revealed a functional impact of the UPR in most hallmarks of cancer (52). However, the significance of UPR signaling at MAMs has not yet been explored in the context of cancer progression. Thus, cancer cells may depend on the structural integrity of MAMs to maintain and balance energy demands, in addition to cope with proteostatic alterations associated with cellular transformation and aggressiveness of tumors. Since XBP1s’ levels are associated with poor prognosis in different tumors (54–56), the stabilization of IRE1 signaling in MAMs is expected to contribute to cancer progression (25), a hypothesis that remains to be tested.

## Concluding Remarks

Interorganelle communication is emerging as a homeostatic network determining the switch from adaptive programs to cell death under stress conditions, where specialized sentinels are localized at organelle membranes to induce the core apoptosis pathway (57). Mitochondria represent an ancestral integrator of stress signals, modulating metabolic demands on a constantly fluctuating environment (58). Although the literature is still poor in relating the activity of the UPR to mitochondrial function, a new model is emerging where proteostasis and metabolic control are tightly interconnected at the structural and functional levels (Figure 2A). This integration might be particularly relevant in pathological conditions such as diabetes and cancer, where the ER and mitochondria undergo high metabolic demands (3). The physical and functional relation between the ER and mitochondria has pleiotropic consequences to the cell by regulating autophagy, ROS production, metabolism, and protein synthesis. At the intersection of all these processes, calcium mobilization is considered a key player in the dynamic cross talk between the ER and mitochondria. Importantly, different core members of the UPR are highly mutated in cancer, suggesting a direct contribution to disease initiation (59). Several pharmacological agents are available to target the UPR with interesting protective effects in cancer (60, 61). It remains to be determined whether these therapeutic agents influence mitochondrial function through MAMs. Overall, the relevance of the intersection between ER and mitochondria is gaining increasing attention in recent years, and thus the specific activities of the UPR at MAMs needs to be systematically studied. Strategies to dissect and manipulate compartmentalized UPR responses may generate novel therapeutic insights, expanding the avenues in the area of drug discovery.

## Author Contributions

AC-S, PP, and CH contributed to the designing and writing of the text.

## Conflict of Interest Statement

The authors declare that the research was conducted in the absence of any commercial or financial relationships that could be construed as a potential conflict of interest.
